# Instructing somebody else to act: motor co-representations in the instructor

**DOI:** 10.1098/rsos.230839

**Published:** 2024-01-10

**Authors:** Mathias Van der Biest, Rebecca Pedinoff, Frederick Verbruggen, Marcel Brass, Anna K. Kuhlen

**Affiliations:** ^1^ Department of Experimental Psychology, Faculty of Psychology and Educational Sciences, Ghent University, Ghent, Belgium; ^2^ Berlin School of Mind and Brain/ Department of Psychology, Humboldt University of Berlin, Berlin, Germany; ^3^ Institute of Psychology, RWTH Aachen University, Aachen, Germany

**Keywords:** instructing, social cognition, instruction-based reflexivity, motor representations

## Abstract

Instructions enable humans to perform novel tasks quickly. This is achieved by creating and activating the instruction representation for upcoming tasks, which can then modulate ongoing task behaviour in an almost ‘reflexive’ manner, an effect called instruction-based reflexivity. While most research has focused on understanding how verbal instructions are represented within the ‘instructed’ (i.e. the person receiving instructions), here we focus on how the instructor's (i.e. the person giving instructions) behaviour is affected through instructing. In a series of three experiments and one pooled analysis, we extended the classical instruction-based reflexivity paradigm to a novel social variant in which the instructions are given by an instructor (rather than visual computer-generated instructions). We found an instruction-based reflexivity effect for the instructor, that is, the instructor's task performance was better on congruent compared to incongruent trials (i.e. Experiments 1 and 2, pooled analysis). This suggests that the instructor represents the instructions of the instructed in an action-oriented format. However, this did not depend on the specific task of the instructed (i.e. Experiment 1), nor is it exclusively social (i.e. Experiment 3).

## 1. Introduction

Humans have the unique capacity to instantly learn new behaviours based on verbal or written instructions [1,2]. For example, in order to learn how to drive, the driver and the instructor must communicate (e.g. turn left), plan (e.g. look in the mirror, activate the blinker, turn the steering wheel), after which the driver must correctly execute the instructions (e.g. execute the planned and communicated behaviour) within seconds to safely make the manoeuvre. This complex ability distinguishes humans from other primates, who take months, if ever, to learn new behaviour (e.g. [3,4]). Indeed, this form of prosocial behaviour has been suggested as one of the driving factors behind human cultural cognition [5], and is a key communication tool [2]. Here, we explore the social dimensions of ‘rapid instructed task learning’ (i.e. RITL) [1,6–8]. We investigated which consequences come for the instructor's own actions when instructing another agent. In other words, whether instructing leads to the representation of the instructions of the others and the activation of the (irrelevant) motor codes when encountering the instructional stimulus.

Based on verbal instructions, we can associate any motor behaviour (i.e. response) with any object (i.e. stimulus), even when we have no experience with the action or object (e.g. [9–11]). This stimulus-response mapping tends to be automatically activated upon being exposed to the stimulus and this from the first encounter [12,13]. Once activated, these stimulus-response mappings can facilitate or interfere with ongoing task behaviour [11,13,14], even when the instructions are no longer relevant [11]. Brass *et al.* [9] found evidence for interference-related activation in the pre-SMA cortex for verbally instructed task sets, even when these were never executed. Similarly, there is evidence for the activation of motor codes in the primary motor cortex without practise [15], and increased lateralized readiness potentials on task-irrelevant dimensions [16]. This suggests that we prepare for the execution of verbal instructions in a reflexive manner (e.g. [17]). Thus, we prepare to implement verbal instructions in the future, and as soon as this intention is formed, the motor responses are (semi-)automatically activated upon encountering the stimulus ([7,11,12,14], but also see [18]). In order to prepare for an upcoming task, we first store the semantics of the instructions in a declarative format and transform them into an action-oriented format (i.e. including all the motor actions to execute the instructions), a process called proceduralization, which is critical for efficient performance (e.g. [6,14]). Once verbal instructions are transformed into an action-oriented format, this can lead to a cognitive phenomenon called ‘instruction-based reflexivity’ [7,8,14,19–22]. Here, motor actions are activated reflexively, independent of the task, stimulus characteristics or action familiarity, and based solely on verbal instructions (e.g. [13,21]).

One experimental task to investigate this reflexivity effect is the IBR task [7,21]. In this nested procedure with a diagnostic and an inducer task, participants first receive two S-R mappings (e.g. dog – left, cat – right) for the inducer task. In this inducer task, participants see one of the two instructed words and press the corresponding button (e.g. if the word ‘dog’ is presented, participants must press the left key). Crucially, prior to the inducer task, participants complete another, nested diagnostic task. In this diagnostic task, participants are presented with the same stimuli (e.g. dog, cat), but respond to a different stimulus dimension. For example, the font of the word (e.g. if the word is printed upright, press left; if the word is printed in italics, press right). This leads to congruent (i.e. cat – italics, dog – upright) or incongruent (i.e. cat - upright, dog - italics) trials. Traditionally, participants respond faster and make fewer errors in congruent compared to incongruent trials. This effect is (mostly) found when there is an intention to implement the instruction. In other words, at some point in the future, the instructions need to be executed. When the inducer task consists of a memorization task, for example indicating whether the presented stimulus-response mapping is the same as the instructed mappings, the effect is (mostly) absent ([21]; but see also [18]). This demonstrates the different instruction representations, namely declarative representations (i.e. memorization) and action-oriented representations (i.e. implementation), and the necessity of proceduralization (i.e. transformation from declarative to action-oriented representations) for the efficient processing of novel instructions.

These studies suggest that (verbal) instructions for a future task are sufficient to form stimulus-response representations that are represented in the procedural working memory. Overall, we have a good understanding of how the person receiving the instructions (the instructed) prepares, represents and implements novel behaviour based on instructions. However, here we shift our object of interest, from ‘*the instructed’* to understanding if and how ‘*the instructor’* prepares, represents and implements the given instructions. Most research on instruction proceduralization is carried out in an individual context with visual computer-generated instructions (e.g. [6,19,21]), thereby ignoring the inherently social and communicative nature of instructions (but see also [8]). This latter aspect is surprising, as previous research has shown that, for example, the trustworthiness of our interaction partner influences advice following [8,23], investments [24], and task cooperation [25]. Thus, investigating instructional processes in a social setting with multiple agents (i.e. instructed and instructor) is critical to advancing our understanding of this unique human skill. In the current set of experiments auditory instructions were exchanged between two active ‘agents’. Specifically, we modified the IBR paradigm to a social variant (i.e. Experiment 1). In this task, an instructor heard two S-R mappings (i.e. inducer instructions). The instructor then instructed a second player, the instructed, who had to execute the inducer task. Before the task of the instructed started, the instructor performed the diagnostic task. This allowed us to look at proceduralization effects on the agent providing the instruction (i.e. instructor), even though the instructor never had to implement the instructed action. It was emphasized that in order to successfully complete the task (i.e. correct response on the inducer probe), the instructor and instructed had to cooperate efficiently even though they were responsible for separate tasks (i.e. instructor: diagnostic, instructed: inducer task), and were therefore judged according to ‘team’ performance (i.e. the successful execution of the inducer task). Such game-like elements were added to promote a sense of collaboration, as only the best performing teams would win an additional bonus (i.e. [25,26]). This was not only emphasized prior to the experiment, but also during the IBR task: after each run of the IBR task, participants received feedback on the performance of the instructed (i.e. inducer task). The best performing team received a voucher (i.e. Experiment 1, 25 euro for each player) and the best seven (i.e. 15%) performing teams in Experiment 2 (i.e. only the participant received 5 euro, not the collaborator, see methods Experiment 2) and Experiment 3 (i.e. both players received a coupon of 5 euro).

In Experiment 1, we investigated whether the IBR effect depends on the task of the executor (i.e. instructed), that is, whether the instructed had to memorize (i.e. declarative working memory, before proceduralization) or implement (i.e. action-oriented representation, after proceduralization) the instructions [21]. Therefore, we wanted to establish under which task conditions the reflexivity effect occurs. We hypothesized that we would find an IBR effect in the condition where the instructed had to implement the instructions (i.e. implementation), but not in the condition where there was no intention to implement, where the instructed only to memorize the instructions (i.e. memorization). In Experiment 2, we ran a preregistered replication of the implementation condition, to ensure that the observed IBR effect in the social setting was not a false positive. In Experiment 3, we investigated whether the IBR effect could also be found in a non-social individual context, in which the instructor simply repeated the instructions aloud without another agent executing the instructions (i.e. no instructed, no inducer task). Lastly, we conducted a pooled analysis, to confirm the IBR effect in a social setting.

## 2. Experiment 1

The goal of Experiment 1 was to establish if the instructor proceduralizes the instructions they give to their task partner, and whether this depends on the to-be-executed task (i.e. memorization versus implementation).

### 2.1. Methods

#### 2.1.1. Participants

Forty-eight pairs of participants (male = 40, female = 56) aged between 18 and 34 years, were invited to the laboratory. All participants were recruited via the Humboldt-Universität zu Berlin's participant recruitment system. We checked for the following exclusion criteria (i.e. diagnostic task, instructors): Participants with an accuracy lower than 60% would be removed from the analyses [8]**,** and participants with a mean reaction time below or above 1.5 IQR (i.e. interquartile range) from the 25th or 75th quantile, would be considered an outlier. None of the participants met these criteria, so the final sample size was *n* = 48. The study was approved by the local ethics committee of the Psychology Department at the Humboldt University of Berlin, and all participants signed an informed consent form.

#### 2.1.2. Materials

The audio files (i.e. 112 stimulus words, and two locations left and right) for the instructing phase and the text stimuli (black Arial 15 pt) for the diagnostic phase were German translations (i.e. 112 words) of black and white line drawings adapted from Snodgrass and Vanderwart, [27]. These images were used as the inducer probe (i.e. 112 images). All stimuli were presented in the centre on a white background. The experiment was programmed in TScope 5 [28] and participants responded using an AZERTY keyboard (i.e. implementation: left or right arrow; memorization: 1/0 number pad).

#### 2.1.3. Design and procedure

This novel, social variant of the IBR task implemented two factors: (1) task condition (memorization versus implementation), which was manipulated between subjects, and (2) task congruency (congruent, incongruent with respect to the instructed task), which was varied within subjects. Participants were randomly assigned to the role of the instructor or instructed. It was emphasized that this was a collaborative task and that they would only succeed at the task by working together. The goal of the task was to gain as many points as possible, by responding correctly on the inducer probe, with the highest scoring team receiving an additional bonus. Thus, only if the instructor provided the correct instructions to the instructed could they correctly execute the inducer task.

The experiment consisted of five phases. In the first phase (i.e. receiving instructions), the instructor received two S-R mappings (e.g. cat – left, dog – right) through headphones (in German). The order of the spatial location (i.e. Stimulus-left/Stimulus-right, or Stimulus-right/Stimulus-left) was randomized. In the second phase (i.e. instructing), the instructor had 3000 ms to verbally instruct the instructed by repeating aloud the two S-R mappings. In the third phase (i.e. diagnostic task), the diagnostic task of the IBR paradigm was executed. During this task, the instructor had two seconds to respond to the stimuli (e.g. dog or cat), with a 750 ms inter-trial-interval. The instructor had to press the left key if the word was printed upright, or the right key if the word was printed in italics. After four or eight trials, the word ‘change’ was presented for 3000 ms, indicating that the task of the instructed would start. The variability in the trial length was randomized and introduced to make the introduction of the inducer task more unpredictable. This resulted in an average of six diagnostic trials per run, and on average 485–486 diagnostic trials per participant. During the fourth phase (i.e. inducer task), the instructed had to react to a probe within a 2000 ms interval. In the implementation condition this consisted of an image of one of the words requiring the instructed to press the corresponding key (i.e. left, or right arrow). In the memorization condition the probe consisted of an image of one of the words paired with either the correct or incorrect response requiring the instructed to indicate whether the stimulus-response mapping was correct or incorrect by pressing 1 (i.e. yes) or 0 (i.e. no). Immediately following the inducer task, both players received feedback (i.e. feedback phase) on the task performance of the instructed. This consisted of the number of points (i.e. the total score: increased by 10 when the response was correct, no change when the response was incorrect), the response of the instructed (i.e. implementation: left or right arrow, memorization: yes or no), and an indication if the response was correct (i.e. correct, or incorrect). During the experiment, the participants received the same computer output. For example, both the instructor and the instructed saw the stimuli (but not the response of the players) of the diagnostic task, the inducer task, and the feedback.

**Figure 1.**
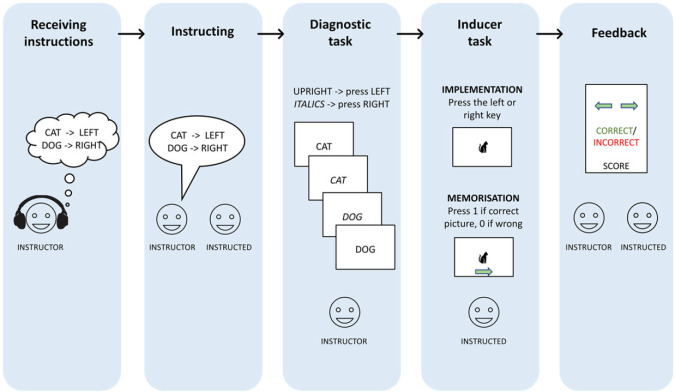
Outline of the experimental procedure for the implementation and memorization conditions. Graphical depiction of one trial. The instructor receives two S-R mappings via a headset (i.e. receiving instructions). Next, the instructor instructs the instructed, who will have to memorize the instructions (i.e. instructing) and execute them when the inducer probe appears (i.e. inducer task). In the meantime, the instructor executes 4 or 8 trials of the diagnostic task (i.e. diagnostic task). Lastly, both agents receive feedback on the performance of the inducer trial. The feedback consisted of the response on the inducer trial, whether this response was correct or incorrect, and the team score.

This sequence was repeated 81 times, divided over three blocks (figure 1). The task was conducted in German.

#### 2.1.4. Preprocessing and analyses

The preprocessing criteria outlined below were applied to all conducted experiments and only to the diagnostic trials of the instructor. Preprocessing of all experiments was conducted in R [29] and analyses were performed in JASP [30]. All analyses scripts and data are available on OSF (https://osf.io/7j93k/).

All correct trials with a reaction time below 200ms (0%), as well as all correct trials where the reaction time deviated 3 standard deviations (i.e. s.d.) from the participant's mean, were considered outliers, and removed from the data (2%). Lastly, all trials following an error were removed (2%). Note that we did not remove trials where the instructor made a mistake during instructing, as we had no written or audio recordings of them.

We conducted separate repeated measures ANOVAs (type III) for reaction times (i.e. RT), error rates (i.e. ER)^1^, and the inverse efficiency scores (i.e. IES) of the diagnostic task. This latter measurement was calculated by dividing the RT by the accuracy (i.e. ACC) and corrects for potential speed-accuracy trade-offs [31]. The IES (i.e. RT/ACC) was included for exploratory purposes, thus, the main measurements of interest were the RT and ER. Each model had congruency as a within-subject factor (i.e. congruent, incongruent) and task condition (i.e. implementation, memorization) as a between-subject factor. For each model, we visually inspected the quantile-quantile and density plots, to check the normality assumption (e.g. [32]). If this assumption was violated for RT or IES, we applied a logarithmic transformation [33,34]. For the ER, we transformed the data with the logit transformation [35].

Post hoc and in addition to the frequentist analyses, we calculated the equivalent Bayes factor for the reported main and interaction effects. All Bayes factors were calculated in JASP [30] with the default priors [36], and interpreted according to the guidelines of JASP [37].

### 2.2. Results: diagnostic task (instructor)

#### 2.2.1. Reaction times

**Figure 2.**
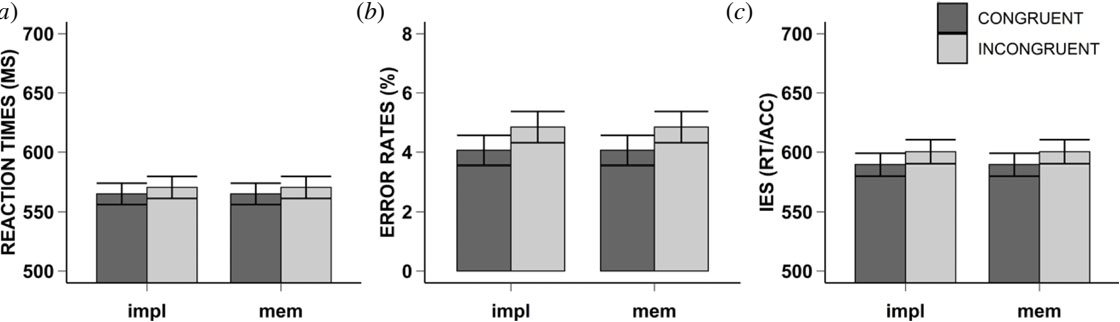
Results Experiment 1. Mean reaction times (i.e. Graph A), error rates (i.e. Graph B), and inverse efficiency scores (i.e. Graph C) for Experiment 1. The bar plots are depicted for each task condition (x-axis): implementation (i.e. impl) and memorization condition (i.e. mem), and congruency (i.e. Congruent: dark grey, Incongruent: light grey). The error bars represent the standard error.

Participants responded significantly faster on congruent (*M* = 565 ms, s.d. = 62) compared to incongruent (*M* = 570 ms, s.d. = 64) trials, *F*_1,46_ = 5.55, *p* = 0.023, $\eta_{p}^{2}=0.11$, BF_10_ = 2.28 (anecdotal evidence for the alternative hypothesis). However, there was no significant interaction between congruency and task condition *F*_1,46_ = 0.06, *p* = 0.816, $\eta_{p}^{2}=0.001$, BF_01_ = 3.28 (moderate evidence for the null hypothesis). Likewise, there was no significant main effect *F*_1,46_ = 0.48, *p* = 0.490, $\eta_{p}^{2}=0.01$, BF_01_ = 1.24 (anecdotal evidence for the null hypothesis). Figure 2*a* and Appendix A for descriptives and density plots.

#### 2.2.2. Error rates

Since the raw data violated the normality assumption, we corrected the data as specified in the Preprocessing and analyses section. Participants made significantly fewer errors on congruent (M = 4%, SD = 4) compared to incongruent trials (M = 5%, SD = 4), *F*_1,46_ = 7.22, *p* = 0.010, $\eta_{p}^{2}=0.14$, BF_10_ = 4.47 (moderate evidence for the alternative hypothesis). There was no significant interaction effect of congruency and task condition *F*_1,46_ = 0.51, *p* = 0.480, $\eta_{p}^{2}=0.01$, BF_01_ = 2.91 (anecdotal evidence for the null hypothesis), nor was there a significant main effect of task condition *F*_1,46_ = 3.36, *p* = 0.073, $\eta_{p}^{2}=0.07$,BF_10_ = 1.19 (anecdotal evidence for the alternative hypothesis). Figure 2*b* and Appendix A for descriptives and density plots.

#### 2.2.3. Inverse efficiency scores

The IES was significantly lower on congruent (*M* = 589, s.d. = 65) compared to incongruent (*M* = 600, s.d. = 70) trials, *F*_1,46_ = 9.94, *p* = 0.003, $\eta_{p}^{2}=0.18$, BF_10_ = 12.06 (strong evidence for the alternative hypothesis). There was no significant interaction effect of congruency and task condition, *F*_1,46_ = 0.12, *p* = 0.727, $\eta_{p}^{2}=0.003$, BF_01_ = 3.10 (moderate evidence for the null hypothesis), nor was there a significant main effect of task condition, *F*_1,46_ = 1.57, *p* = 0.216, $\eta_{p}^{2}=0.03$, BF_01_ = 1.60 (anecdotal evidence for the null hypothesis).

### 2.3. Discussion

Experiment 1 demonstrated that instructors proceduralized the instructions they gave to their task partner. This was shown for both RT, ER and IES. Our Bayesian analyses indicated moderate (ER) and strong (IES) evidence for the IBR effect, and anecdotal evidence for the RT. Overall, the results of Experiment 1 suggest that the instructor represents the stimulus-response mappings of the instructed, even though these are irrelevant to their own task. Furthermore, the instructions are represented in an action-oriented format within the instructor, as reflected by the IBR effect for all dependent measures. This is in line with work demonstrating IBR effects for the instructed (e.g. [11,14,21]), namely, that there is a reflexive preparation for task implementation [7,11,12,14,17,18], and that automatic activation of the motor code modulates the performance of ongoing task behaviour [14].

Contrary to our predictions, we found no significant difference between the memorization and implementation conditions, suggesting that the to-be-executed task of the instructed does not modulate the IBR effect. Our Bayesian analyses indicated anecdotal (ER) to moderate (RT, IES) evidence for the null hypothesis. This suggests that even when the instructed only represent the instructions in a declarative manner, and thus when one would not expect an IBR effect [21], the instructor still proceduralized the instructions. And thus, that the act of instructing another agent leads to an action-oriented representation of the instructions, regardless of the exact nature of the to-be-executed task.

## 3. Experiment 2

In Experiment 2, we aimed to replicate Experiment 1 by means of a preregistered study (https://aspredicted.org/xr8dn.pdf), with an optimized version of the social IBR paradigm, in a different language (i.e. Dutch) and with an increased sample size (*n* = 48 instead of 24 in the implementation condition). As there was no significant difference between the memorization and implementation condition in Experiment 1, we only included the latter condition. Furthermore, we made the experimental task more similar to the paradigm used in traditional IBR research (e.g. number of diagnostic trials, blocks, words instead of images in the inducer task) (e.g. [8,14,21]). Finally, we extended the experiment by including a non-social individual condition in which the instructor executed the same task but without the presence of the instructed. Unfortunately, due to a technical error during data collection, we could not include this condition in the analyses (which is why we included this condition in Experiment 3; see below).

### 3.1. Method

#### 3.1.1. Participants

Fifty participants (35 females, 15 males, Mage = 20.2, SDage = 5.4) were recruited from the SONA recruitment platform from Ghent University and participated in exchange for either a course credit or monetary compensation. This study was preregistered, but in the end, we applied different exclusion criteria than the preregistered ones in order to standardize all experiments reported in this manuscript. The analyses according to the preregistered criteria can be found in the supplementary materials. The conclusions are not influenced by the exclusion criteria. Specifically, we applied a different outlier criterion for reaction times. As in Experiment 1, participants were excluded if their mean reaction times were 1.5 IQR below the 25th or above the 75th percentile from the group median. We decided to apply this criterion instead of the preregistered criterion (i.e. 3SD above or below the mean) as the latter might be suboptimal to detect outliers at the participant level (e.g. [38,39]). Two participants were excluded because of too many mistakes on the diagnostic task (i.e. ACC < 60%), and one participant was excluded because they responded too slowly (i.e. 1.5IQR above the 75th percentile). Given that this latter exclusion criterion was only implemented after we finished data collection, the final sample deviates from the preregistration (i.e. 47 instead of 48, 32 females, 15 males, Mage = 20.2, SDage = 5.5). We reasoned that doubling the sample size of the implementation condition for Experiment 1 would provide sufficient power to detect a small to medium effect (*d* = 0.40; [40]). The experiment was conducted in accordance with the local institutional ethics committee of Ghent University, and all participants gave written informed consent.

#### 3.1.2. Materials

For the audio instructions, we recorded 102 Dutch four-letter nouns that we matched for word frequency [41]. These were identical to the words used by Van der Biest *et al.* [8]. All stimuli were presented on a 15-inch Dell computer monitor (i.e. P2419H, instructor) on a black background with a white font (i.e. cues and stimulus Arial 15). The audio instructions were presented through Sennheiser 215 headphones. The experiment was programmed in PsychoPy (v2021.1.2.3) and Python (3.6.6) [42].

#### 3.1.3. Design and procedure

Participants were invited to the laboratory and chose one of two envelopes to decide who was the instructor and who was the instructed. Crucially, the participants were always assigned the role of instructor, as both envelopes contained the same player assignment. The data were collected during the COVID-19 pandemic, in accordance with the health guidelines of the Belgian government and Ghent University. Therefore, the instructed was a confederate (i.e. the experimenter and first author of this paper). Following, the tasks of the instructor (i.e. participant) and the instructed (i.e. confederate) were explained, and it was emphasized that the best performing 15% of all teams would win an additional voucher (i.e. in fact this was only for the participant, see also Design and procedure Experiment 1). The instructor was informed that only when they provided the correct instructions would the instructed be able to respond correctly to the inducer probe, and efficiently execute the task.

The phases of the experiment were the same as in Experiment 1, namely, receiving the instructions, instructing, the diagnostic task, the inducer task and feedback. However, the order of the spatial location (i.e. 50% of the trials Stimulus-left/Stimulus-right, and 50% of the trials Stimulus-right/Stimulus-left) was now counterbalanced (see Design and procedure Experiment 1). In a similar vein, the number of trials for the diagnostic run (i.e. the diagnostic task) was 4, 8, 12 or 16 instead of only 4 or 8 (i.e. Experiment 1), and this was counterbalanced within blocks. During the inducer task, the instructed had to respond to one of the two words (i.e. on 50% this was the left word, on 50% the right word) by pressing the left or right arrow, instead of an image of one of the two words. And the experimental sequence was repeated eight times for six blocks, instead of 27 times for three blocks (i.e. Experiment 1). Prior to the experimental procedure (figure 1), there were two practice trials. Please note that during the practice runs, the instructor also received feedback (i.e. red square if a mistake was made, both players received the feedback). As with Experiment 1, both players saw the exact same computer output during the experiment.

The design was within-subject with congruency as an independent variable (i.e. congruent, incongruent).

#### 3.1.4. Preprocessing and analyses

Data preprocessing was almost identical to Experiment 1, except that all practice trials and trials on which the instructor made a mistake when instructing, were removed from the analyses (7%). Due to our RT outlier criteria at trial level, 2% of all correct trials were considered outliers (i.e. less than 0.0001 of the trials were faster than 200ms or no response was given; 2% were ±3s.d. from the participant's mean) [43]. Lastly, 3% of all trials were removed as they were preceded by an error (e.g. [8]). The discussed trial removal criteria deviated from the preregistered criteria. This is because we realized afterwards that the preregistered criteria were prone to increased Type I error due to the removal of trials for each condition, rather than the whole dataset [44]. The deviations did not affect our conclusions, indicating that the findings reported below are robust. See the supplementary materials for the analyses following our preregistation.

To test our hypotheses, we constructed a repeated measures ANOVA (type III) for each dependent variable (i.e. RT, ER, IES) with congruency (i.e. congruent, incongruent) as a factor for the diagnostic task (i.e. instructor). Like Experiment 1, we calculated the Bayes factor (see Preprocessing and analyses of Experiment 1 for procedure).

### 3.2. Results: diagnostic task (instructor)

#### 3.2.1. Reaction times

**Figure 3.**
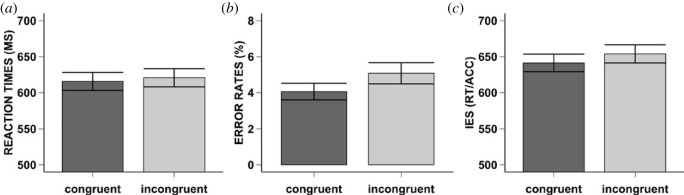
Result Experiment 2. Mean reaction times (i.e. Graph A), error rates (i.e. Graph B), and inverse efficiency scores (i.e. Graph C) for Experiment 2. The error bars represent the standard error. The bar plots are separate for each condition of congruency (i.e. Congruent: dark grey, Incongruent: light grey).

We found a numerical difference between congruent (*M* = 615 ms, s.d. = 85) and incongruent (*M* = 621 ms, s.d. = 87) trials. However, this was not significant *F*_1,46_ = 2.60, MSE = 253.98, *p* = 0.114, $\eta_{p}^{2}=0.05$, BF_01_ = 1.55 (anecdotal evidence for the null hypothesis). Figure 3*a* and Appendix A for density plots.

#### 3.2.2. Error rates

For the error rates, the normality assumption was violated (see Experiment 1: Preprocessing and analyses, for procedure). After correction, we found that participants made significantly fewer errors on congruent (*M* = 4%, s.d. = 3) compared to incongruent trials (*M* = 5%, s.d. = 4), *F*_1,46_ = 6.27, MSE = 0.07, *p* = 0.015, $\eta_{p}^{2}=0.12$, BF_10_ = 2.99 (anecdotal evidence for the alternative hypothesis). Figure 3*b* and Appendix A for density plots.

#### 3.2.3. Inverse efficiency scores

Lastly, our exploratory analyses revealed that the IES was significantly lower in congruent (*M* = 641, s.d. = 85), compared to incongruent (*M* = 654, s.d. = 86) trials, *F*_1,46_ = 7.01, MSE = 531.85, *p* = 0.011, $\eta_{p}^{2}=0.13$, BF_10_ = 3.90 (moderate evidence for the alternative hypothesis). Figure 3*c* and Appendix A for density plots.

### 3.3. Discussion

The results of Experiment 2 confirm our initial hypothesis, at least for the ER, as participants made fewer errors on congruent compared to incongruent trials (i.e. anecdotal evidence for the alternative hypothesis), and for the IES (i.e. moderate evidence for the alternative hypothesis), which was significantly lower for congruent than for incongruent trials. There was no significant congruency effect for reaction times (i.e. anecdotal evidence for the null hypothesis), although the small numerical difference was in the same direction as Experiment 1. Thus, the irrelevant instructions for the inducer task (i.e. task of instructed) influence the performance during the diagnostic task (i.e. instructor). It is noteworthy, that the reported effect sizes (medium to strong) in Experiments 1 and 2 slightly differ from the traditional effect (strong) sizes within the IBR paradigm, where one participant performs both the diagnostic and inducer task [8,14,45,46]. As the participants performs both tasks, the inducer instructions are more relevant, which potentially results in stronger action-oriented representation, as measured with larger differences between congruent and incongruent trials.

Crucially, it is not only important to show that this effect is present in a social condition, but also that this effect is absent in a non-social individual condition. If one were to find such IBR effects in a non-social individual condition, where there is no one executing the instructions and thus no one being instructed, this would suggest that the formation of the instruction representation is not (entirely) due to the social context but would indicate that other non-social processes might play a role. For example, it could be that hearing the instruction, since the instructor always receives instructions auditorily, or verbalizing the instruction (i.e. repeating it aloud) leads to the representation of the instruction.

## 4. Experiment 3

To investigate the social nature of our findings, we conducted a third study. In line with the joint action literature (for a review see [47]), we directly compared the IBR effect in a social with a non-social individual condition (e.g. [48–51]).

In this within-subject study, the instructor either instructed another agent who executed the instructions (i.e. Experiments 1 and 2), or performed the experiment alone (i.e. no agent executing the instructions). We expected an IBR effect in the social (i.e. Experiments 1 and 2), but not in the non-social individual condition.

### 4.1. Method

#### 4.1.1. Participants

We recruited 59 pairs of participants (Mage = 20.59, SDage = 4.56, 26 males, 93 females) from the SONA recruitment platform of Ghent University. Participants were either first-year psychology students who received one course credit for their participation or participants who received a 10-euro reimbursement. One participant was excluded because of our accuracy criteria (i.e. ACC < 60%), another participant was excluded because they responded too slowly (i.e. 1.5IQR from the 75^th^ percentile), and nine participants were excluded due to repeating the instructions too slowly, making too many errors when repeating, or not repeating the instructions at all. This led to a total sample of 48 (Mage = 20.50, SDage = 5.10, 13 males, 45 females). This preregistration can be found here: https://aspredicted.org/h2q5k.pdf. The experiment was conducted in accordance with the local institutional ethics committee of Ghent University, and all participants gave written informed consent.

#### 4.1.2. Materials

The task and materials were almost identical to Experiment 2. The crucial difference was that participants executed a social IBR task (i.e. with two players, see Experiments 1 and 2), and also a non-social individual IBR task (i.e. only one player). We created a new set of audio recordings consisting of 102 Dutch four-letter nouns matched for word frequency [41]. In addition, we used the same audio recordings from Experiment 2 and randomly combined the 204 Dutch words into two sets. The voice was identical for both sets. The experiment was programmed in PsychoPy (v2021.1.2.3) and Python (3.6.6) [42] and run on a Dell 2419H screen in three separate rooms (i.e. one for the social condition, and two for the individual condition) at Ghent University. All stimuli were presented on a black background and printed in white. The instructor wore Sennheiser 215 headphones.

#### 4.1.3. Design and procedure

Two participants (i.e. instructor and instructed) were invited to the laboratory. One of the participants was randomly assigned as the instructor, and the second participant as the instructed. It was emphasized that the best performing (i.e. best 15%) pairs would receive an additional voucher of ten euros (i.e. each 5 euro). As in Experiments 1 and 2, it was emphasized that the participants had to work together, and that only if the instructor provided the correct instructions to the instructed could the task be performed efficiently and correctly (i.e. see Design and procedure Experiment 1,2).

Half of the participants started with the social condition, and the remaining half with the non-social condition. The social condition was identical to Experiment 2: firstly, the instructor heard the instructions, instructed the other participant (i.e. instructed), and executed the diagnostic task. The instructed participant then executed one trial of the inducer task. For the non-social condition, there were two main differences. First, the experimental task was executed individually (i.e. no instructed), and secondly, the repeated instructions were never executed (i.e. no inducer task). In other words, in the non-social condition, both participants, separate from each other, executed the experiment as instructors following the identical procedure and apparatus as with the social condition except for the absence of the inducer task. More information about the number of trials, timing, feedback, and response time can be found in the Design and procedure section of Experiments 1 and 2.

Overall, the experiment had a 2×2 within-subject design, with congruency (i.e. congruent, incongruent) and socialness (i.e. social, and non-social) as independent variables.

#### 4.1.4. Preprocessing and analyses

The preprocessing procedure was identical to that used in the previous studies (i.e. Experiments 1,2). If the instructor made an error when repeating the instructions, all trials from that diagnostic run were removed from the analyses (5%). All correct trials faster than 200 ms (less than 0.0001%), or which were 3SD from the participant's mean were considered outliers (2%). Lastly, all trials following an error were removed from the data (3%).

We performed a repeated measures ANOVA (type III) with congruency (i.e. congruent versus incongruent) and socialness (i.e. social versus non-social) as factors for RT, ER and IES. We did not analyse the collected data of the individual condition for the instructed, as we were only interested in the task performance of the instructor. We calculated the Bayes factor as well (i.e. post hoc, see Preprocessing and analyses Experiments 1 and 2).

### 4.2. Results: diagnostic task (instructor)

#### 4.2.1. Reaction times

**Figure 4.**
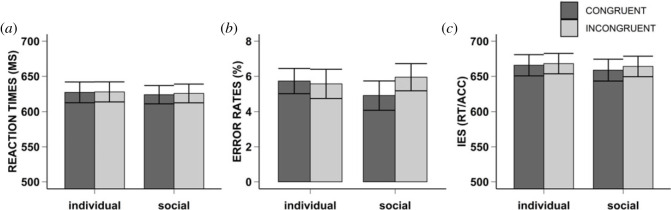
Results Experiment 3. Mean reaction times (i.e. Graph A), error rates (i.e. Graph B), and inverse efficiency scores (i.e. Graph C) for Experiment 3. The error bars represent the standard error. The bar plots are separate for each condition of congruency (i.e. Congruent: dark grey, Incongruent: light grey), and socialness (i.e. individual, and social).

For the analyses of the reaction times, we corrected for violations of the normality assumption (see Experiment 1: Preprocessing and analyses for procedure) and found no significant main effect of socialness *F*_1,47_ = 0.87, MSE < 0.001, *p* = 0.356, $\eta_{p}^{2}=0.02$, BF_01_ = 3.10 (moderate evidence for the null hypothesis), nor a significant main effect of congruency, *F*_1,47_ = 0.07, MSE < 0.001, *p* = 0.800, $\eta_{p}^{2}=0.001$, BF_01_ = 3.51 (moderate evidence for the null hypothesis), nor a significant interaction effect, *F*_1,47_ = 0.01, MSE < 0.001, *p* = 0.910, $\eta_{p}^{2}<0.001$, BF_01_ = 5.43 (moderate evidence for the null hypothesis). Figure 4*a* and Appendix A for descriptives and density plots.

#### 4.2.2. Error rates

We corrected the error rates for violations of the normality assumption (see Experiment 1: Preprocessing and analyses for procedure), and found no significant main effect of congruency, *F*_1,47_ = 2.50, MSE = 0.06, *p* = 0.120, $\eta_{p}^{2}=0.05$, BF_01_ = 2.71 (anecdotal evidence for the null hypothesis), nor a significant main effect of socialness, *F*_1,47_ = 2.84, MSE = 0.23, *p* = 0.099, $\eta_{p}^{2}=0.06$, BF_01_ = 2.38 (anecdotal evidence for the null hypothesis), nor a significant interaction effect, *F*_1,47_ = 1.20, MSE = 0.07, *p* = 0.279, $\eta_{p}^{2}=0.03$, BF_01_ = 3.65, (moderate for the null hypothesis). Figure 4*b* and Appendix A for descriptives and density plots.

#### 4.2.3. Inverse efficiency scores

The exploratory analyses of the inverse efficiency scores revealed no significant main effect of congruency, *F*_1,47_ = 1.32, MSE = 527.92, *p* = 0.257, $\eta_{p}^{2}=0.03$, BF_01_ = 3.21 (moderate evidence for the null hypothesis), nor a significant main effect of socialness *F*_1,47_ = 0.50, MSE = 2874.36, *p* = 0.483, $\eta_{p}^{2}=0.01$, BF_01_ = 2.91 (anecdotal evidence for the null hypothesis), nor a significant interaction effect, *F*_1,47_ = 0.20, MSE = 537.35, *p* = 0.657, $\eta_{p}^{2}=0.004$, BF_01_ = 2.68 (anecdotal evidence for the null hypothesis). Figure 4*c* and Appendix A descriptives and density plots.

### 4.3. Discussion

With Experiment 3 we aimed to replicate the findings from Experiments 1 and 2. Moreover, and in line with the joint action literature (e.g. [48–51]), we wanted to establish whether the IBR effect observed in the previous two experiments was indeed due to the social context. Therefore, we directly compared how the instructor performed in a social and in a non-social individual condition in a within-subject design.

Numerically, we found evidence for a greater IBR effect for RT, ER and IES in the social compared to the individual non-social condition. But these differences were small and statistically not significant. This absence of a significant interaction effect (i.e. anecdotal and moderate evidence for the null hypothesis) was not explained by data quality issues, as there were few to no mistakes when instructing the instructed, and numerically the various dependent measurements (i.e. RT, ER, IES) were in line with our previous findings and other studies (e.g. [8,14]).

Given this unexpected finding, we wanted to re-establish the IBR effect observed in Experiments 1 and 2, by running a post hoc pooled analysis on all the collected social data from the implementation condition. This increase in statistical power allowed us to detect smaller differences and have a solid test of IBR effects when instructing another agent.

## 5. Pooled analysis

### 5.1. Method

#### 5.1.1. Analyses

We grouped all the collected data of the social implementation conditions according to congruency (i.e. congruent versus incongruent) and ran an ANOVA (i.e. type III), and equivalent Bayesian tests for each dependent measurement (i.e. RT, ER, IES).

### 5.2. Results: diagnostic task (instructor)

#### 5.2.1. Reaction times

**Figure 5.**
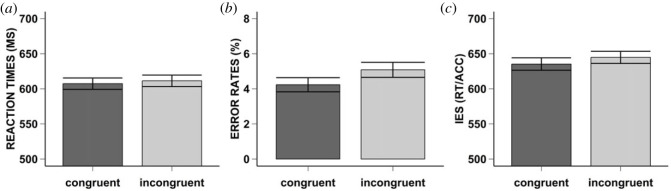
Results pooled analysis. Mean reaction times (i.e. Graph A), error rates (i.e. Graph B), and inverse efficiency scores (i.e. Graph C) for the pooled analysis. The error bars represent the standard error. The bar plots are separate for each condition of congruency (i.e. Congruent: dark grey, Incongruent: light grey).

Participants were significantly faster on congruent (*M* = 607 ms, s.d. = 88), compared to incongruent (*M* = 611 ms, s.d. = 89) trials, *F*_1,118_ = 5.55, MSE = 174.98, *p* = 0.020, $\eta_{p}^{2}=0.05$, BF_10_ = 1.75 (anecdotal evidence for the alternative hypothesis). Figure 5*a* and Appendix A for density plots.

#### 5.2.2. Error rates

The normality assumption was violated. For the corrected error rates (see Experiment 1: Preprocessing and analyses, for procedure), we found that participants made significantly fewer errors on congruent (*M* = 4%, s.d. = 4) compared to incongruent (*M* = 5%, s.d. = 5) trials, *F*_1,118_ = 16.18, *p* < 0.001, MSE = 0.06, $\eta_{p}^{2}=0.12$, BF_10_ = 187.85 (extreme evidence for the alternative hypothesis). Figure 5*b* and Appendix A for density plots.

#### 5.2.3. Inverse efficiency scores

Analyses of inverse efficiency scores revealed that the IES was significantly lower for congruent (*M* = 635, s.d. = 97) compared to incongruent (M = 645, SD = 94) trials, *F*_1,118_ = 13.77, *p* < 0.001, MSE = 392.70, $\eta_{p}^{2}=0.10$, BF_10_ = 67.57 (very strong evidence for the alternative hypothesis). Figure 5*c* and Appendix A for density plots.

### 5.3. Discussion

In our pooled analyses, we combined the collected data of the social implementation conditions to assess whether there was indeed an IBR effect. Our hypotheses were confirmed as we found an IBR effect for RT, ER, and IES, indicating that the instructors perform significantly better (i.e. faster, fewer errors, and with lower inverse efficiency scores) on congruent compared to incongruent trials. This was partially confirmed by the Bayes factor, indicating very strong (i.e. IES), extreme (i.e. ER), and anecdotal (i.e. RT) evidence for the alternative hypothesis.

## 6. General discussion

In the present study, we investigated whether instructing another agent (i.e. instructed) leads to the processing of the instructions and activation of the corresponding motor codes within the instructor. The current set of experiments extended the literature by focusing on instruction-based reflexivity effects within the instructor rather than of the instructed, in a more natural and social context. In Experiment 1, we established an IBR effect for the instructor independent of the task of the instructed (i.e. implementation versus memorization) and replicated this effect for the implementation condition in Experiment 2. In Experiment 3, we evaluated whether this effect could also be found in a non-social context, and to our surprise, we found no difference between the social and individual conditions, nor a congruency effect in general. Given the high accuracy of the instructed on the inducer task (*M* = 97%, s.d. = 2, range = 92–100%), and the low number of mistakes when repeating the instructions (5%), this absence was likely not due to the quality of the stimulus, the motivation of the instructor, or a lack of collaboration. In addition to the reported experiments, we conducted two online pilots testing the IBR effect in an individual condition with slight modifications of the paradigm. In the first pilot with an English-speaking population (*N* = 84), we found an IBR effect on reaction times, but not on error rates nor in the inverse efficiency scores. In the second pilot (*N* = 48), with a Dutch-speaking population and the same stimulus as in Experiments 2 and 3, we did not find evidence for an IBR effect in any of the dependent measures. Although these pilots are beyond the scope of this paper, the results and raw data can be found here (https://osf.io/7j93k/), and may serve as pilot data for future research. Finally, to increase our statistical power, we pooled the data from Experiments 1–3 (i.e. only social implementation conditions), and found consistent IBR effects for reaction times, error rates and inverse efficiency scores in the social settings.

These findings suggest that, similar to instruction implementation within the instructed ([7,11,12,14], but see also [18]), that the instructor processes the semantics of the instructions (e.g. [19]), proceduralizes them [6], and finally represents the instructions in an action-oriented format [20,22], which modulates the ongoing task behaviour [14]. This is consistent with findings from the joint action literature, which showed that we co-represent stimulus-response mappings (e.g. [50]), co-actors actions when clinking a glass [52], when playing music [53], or during word encoding [54] (for a review see [51]). However, contrary to our predictions, we did not find a difference between the social and the individual condition, suggesting that not only social but also non-social processes may play a role.

For example, the absence of the interaction effect is potentially explained by a second type of instructing. In the individual condition, the participant also repeats the instructions, and this action is inherently a form of instructing, namely, self-instructing or self-verbalization. Verbal self-instructing is an established psychological intervention tool [55], and has been shown to reduce task switching costs in children and older adults [56], improve verbal math [57], scientific problem-solving [58], enhance motor performance [59], increase attention in athletes [60] and increase attentional processing of task-relevant features [61]. Thus, by self-instructing the task set mappings of the irrelevant inducer task becomes relevant, and the instructor potentially processes the instructions and forms a preparatory representation of the mappings, resulting in the IBR effect. This self-instructing behaviour can therefore be seen as a self-preparation and attentional mechanism for upcoming tasks.

Alternatively, prior to instructing the other player, the instructor received two stimulus-response mappings on each trial. Although these were originally intended to prompt the instructor to give instructions to the task partner, the act of conveying these instructions to the instructed may have prompted the instructor to process them. Previous research has shown that stable S-R associations can be formed on the basis of verbal encoding, leading to congruency-like effects, irrespective of the execution of an action (e.g. [62]), and theories of embodied cognition propose that the processing of actions (e.g. kicking, clinking…) at a semantic level, is sufficient to activate the corresponding motor or sensory codes (for a review see [63]). However, there is substantial evidence from behavioural (e.g. [7,14,21]), neuroimaging [9,15,19,20], and preparatory motor activation [16] studies indicating the distinction between semantic representations (i.e. declarative working memory) and action-oriented representations (i.e. procedural working memory). In order to transform the declarative into an action-oriented representation (i.e. proceduralization), there must be an ‘intention to implement’ the instructions either immediately or in the future ([11,13,14,21], but see also [18,46]). Similarly, instruction-based reflexivity has an inherently preparatory nature [2,12,13,17]. Therefore, it seems unlikely that merely receiving auditory instructions leads to the active processing and preparation for the execution of the inducer instruction.

Finally, it could be argued that the memorization process after receiving the instructions and before repeating the instructions is the driving factor behind the IBR effect. Indeed, on each trial the instructor must memorize the instructions, as only then can the instructions be repeated correctly. As previously discussed, the majority of studies propose that the intention to implement the instructions is essential for instruction proceduralization (e.g. [9,13,21]). However, there are two studies suggesting that this is not mandatory. Theeuwes *et al.* [46] found an IBR effect for response-effect mappings when the inducer task was a recognition task (i.e. does the effect match the stimulus). Similarly, an IBR effect was also found with stimulus-response mappings in a task similar to the paradigm used in our study [18]. This led to the conclusion that the ‘intention to implement’ is not a prerequisite for finding an IBR effect. Research has shown that motivation and incentives are important mechanisms for improving cognitive performance. For example, monetary incentives enhance visual working memory in children with ADHD [64], influence prefrontal regions associated with working memory and performance on verbal working memory tasks [65], increase performance on intelligence tests [66], and loss-threatening incentives result in fewer errors and faster responses [67], for a review see for example von Bastian & Oberauer [68] or Westbrook & Braver [69]. Given the high relevance of memorization in the task design, the incentive, motivation and effort to memorize the instructions, as only then can the instructor correctly instruct the response-mappings for the inducer task, the instructor might have represented the instructions in an action-oriented format, resulting in the IBR effect. Although the primary aim was to enhance the teamwork experience, the issuance of a reward to the social context may have led to more motivation for the instructor, and thus unintentionally introduced a confound in the social condition (as there is no reward in the individual condition). Future studies should take this into account, and perhaps even empirically investigate whether removing the team reward leads to a reduction or even diminishes the IBR effect in the social condition.

## 7. Conclusion and future directions

In the current study, we modified the classic IBR paradigm [14] to include a social collaborative version. We first found that under certain task conditions, instructing a task partner can lead to the formation of instruction representations on behalf of that partner, which affects one's own performance. To our surprise, we did not find a difference between individual and social contexts, and the IBR effect did not depend on the to-be-executed task. We discuss three potential explanations for our findings and suggest that future studies should focus on comparing the social and individual conditions with an increased sample size. Lastly, future studies should make a dissociation between the ‘repetition of the stimulus', ‘stimulus presentation’ and ‘memorization’ hypotheses.

## Data Availability

All presented results are based on new data, collected by the authors. All data and scripts can be found here: https://osf.io/7j93k/. Electronic supplementary material is available online [70].

## Footnotes

1 We originally reported and preregistered the accuracy, but one reviewer pointed out that the interpretation of the IBR effect is more straightforward when the reaction times and error rates are in the same direction. In all discussed experiments, the error rates are reported.

## Appendix A. Descriptive results for all experiments

**Appendix A:**

experiment	dependent	group	congruent	mean (s.d.)
**Experiment 1**	RT	memorization	congruent	572(50)
			incongruent	577(57)
		implementation	congruent	559(73)
			incongruent	565(72)
	ACC	memorization	congruent	95(4)
			incongruent	94(4)
		implementation	congruent	97(3)
			incongruent	96(4)
	IES	memorization	congruent	602(57)
			incongruent	611(66)
		implementation	congruent	577(71)
			incongruent	589(73)
**Experiment 2**	RT		congruent	615(85)
			incongruent	621(87)
	ACC		congruent	96(3)
			incongruent	95(4)
	IES		congruent	641(85)
			incongruent	654(86)
**Experiment 3**	RT	social	congruent	624(90)
			incongruent	626(92)
		individual	congruent	627(103)
			incongruent	628(99)
	ACC	social	congruent	95(6)
			incongruent	94(5)
		individual	congruent	94(5)
			incongruent	94(6)
	IES	social	congruent	659(108)
			incongruent	664(101)
		individual	congruent	666(105)
			incongruent	668(100)

**Rain Cloud Plot Experiment 1**

*Note.* The black raincloud plots represent the congruent condition, the grey plots the incongruent conditions. Each raincloud plot depicts the raw mean for each participant (dots on the left), the group mean (large dot on the midline), and the interquartile interval (0.05–0.95) as the error bars. Additionally, on the right side of the midline, the density distribution is represented. The upper panel represents the subjects from the implementation condition. Graph A is a depiction of the reaction times in milliseconds. Graph B represents the error rates, and graph C the inverse efficiency scores. The lower panel represents the subjects from the memorization condition. Graph D is a depiction of the reaction times in milliseconds. Graph E represents the error rates, and graph F the inverse efficiency scores.

**Rain Cloud Plot Experiment 2**

*Note.* Results of Experiment 2. The black raincloud plots represent the congruent condition, the grey plots the incongruent conditions. Each raincloud plot depicts the raw mean for each participant (dots on the left), the group mean (large dot on the midline), and the interquartile interval (0.05–0.95) as the error bars. Additionally, the density distribution is represented on the right side of the midline. Graph A represent the rain cloud plot of the reaction times, graph B the error rates, and graph C the inverse efficiency scores.

**Rain Cloud Plot: Experiment 3**

*Note.* Results of Experiment 3. The black raincloud plots represent the congruent condition, the grey plots the incongruent conditions. These are separated for each level of socialness (x-axis: individual, social). Each raincloud plot depicts the raw mean for each participant (dots on the left), the group mean (large dot on the midline), and the interquartile interval (0.05–0.95) as the error bars. Additionally, the density distribution is represented on the right side of the midline. Graph A represents the rain cloud plot of the reaction times, graph B the accuracy, and graph C the inverse efficiency scores.

**Rain Cloud Plot: pooled analysis**

*Note.* Results of the pooled analyses. The black raincloud plots represent the congruent condition, the grey plots the incongruent conditions. Each raincloud plot depicts the raw mean for each participant (dots on the left), the group mean (large dot on the midline), and the interquartile interval (0.05–0.95) as the error bars. Additionally, the density distribution is represented on the right side of the midline. Graph A represents the rain cloud plot of the reaction times, graph B the error rates, and graph C the inverse efficiency scores.
